# Metal ferrite derivative chemical looping systems: a review towards a multiscale approach for technology readiness enabling clean energy conversion and carbon neutrality

**DOI:** 10.1039/d4sc05865h

**Published:** 2025-03-04

**Authors:** Tanay A. Jawdekar, Ishani Karki Kudva, Sudeshna Gun, Shekhar G. Shinde, Ashin A. Sunny, Zhuo Cheng, Liang-Shih Fan

**Affiliations:** a William G. Lowrie Department of Chemical and Biomolecular Engineering, The Ohio State University Columbus OH 43210 USA fan.1@osu.edu

## Abstract

Chemical looping technologies offer a promising pathway for clean energy production, addressing the urgent need for decarbonization in light of escalating global energy demands and climate change concerns. This review explores the metal ferrite oxygen carriers in chemical looping applications, emphasizing their versatility in handling diverse feedstocks—from gases like methane to solids like plastics—and their robust performance in terms of stability and efficiency. The ferrite derivative chemical looping reactions involve the transfer of lattice oxygen from the metal ferrites to the fuel, enhancing fuel conversion without direct emission of pollutants. The structural and functional advantages of ferrites, including their ability to regenerate and sustain repeated redox cycles, are highlighted. Innovations in ferrite-based chemical looping, from small-scale laboratory setups to pilot-scale installations, demonstrate significant advancements in achieving high energy–exergy efficiencies with minimal ecological impact. The review also identifies ongoing challenges, such as the stability and effectiveness of metal ferrite oxygen carriers, suggesting improvements through material engineering and process optimization. This work aims to deepen understanding of ferrite oxygen carriers and propel forward their application in scalable, commercially viable clean energy solutions.

## Abstract 2

Chemical looping technologies offer a promising pathway for clean energy production, addressing the urgent need for decarbonization in light of escalating global energy demands and climate change concerns.

## Graphical abstract

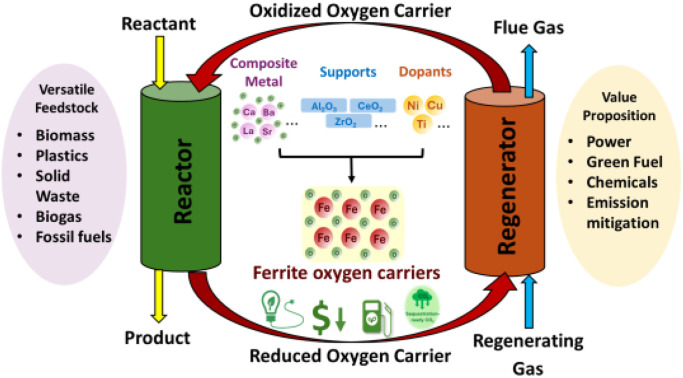

## 1 Introduction

The unprecedented increase in energy demand coupled with a growing concern for climate change has created an impetus for clean energy production.^1^ Although a great emphasis is observed on harnessing energy from renewable sources, the U.S. Energy Information Administration has projected that more than 50% of the world's energy demand will be dependent upon fossil-based energy sources by 2050.^2^ The growing industrial sector consumes ∼33% of the total energy produced in the U.S., which amounts to nearly 26.7 quadrillion British thermal units (BTU).^3^ The production of commodity chemicals like syngas, hydrogen (H_2_), methanol (CH_3_OH), ethanol (C_2_H_5_OH), ammonia (NH_3_), *etc.*, *via* state-of-the-art technologies like auto-thermal reforming (ATR), steam methane reforming (SMR), Haber–Bosch process, *etc.* consumes ∼35% of total industrial energy.^4^ Although these technologies have been established for decades and have proven to be economical at larger scales of operation, they are challenged by carbon deposition and poor catalyst stability, leading to the formation of undesirable side products.^1^ This results in huge capital investment and high operating costs to comply with environmental protection policies. International treaties like the Paris Agreement aim to limit atmospheric CO_2_ concentration below 430 ppm by 2030, necessitating the de-carbonization of industrial processes.^5,6^

Chemical looping (CL) is an emerging versatile alternative to produce commodity chemicals with inherent emission control.^7–10^ This technology involves splitting the reaction scheme into a series of steps, facilitating complete fuel conversion, inherent product separation, and higher energy–exergy efficiencies.^11–15^ Splitting of the reaction scheme is facilitated by a metal oxide carrier circulating within interconnected reactors and undergoing subsequent reduction and regeneration cycles.^16,17^ During the reduction reaction, the metal oxide carrier donates its lattice oxygen to the fuel, resulting in the complete conversion of the fuel to the desired products (carbon dioxide – CO_2_, steam – H_2_O, or carbon monoxide – CO, H_2_). This lattice oxygen is then replenished using oxidizing gases like air, steam, or CO_2_, thus completing the redox loop. The overall reaction scheme remains the same as combustion or reforming.^18,19^

Breaking down the reaction scheme in this manner enables the isolation of the pure products without the need for a separation unit after the reactor.^20^ While breaking the reaction into several steps helps to optimize each individual step to obtain near thermodynamic yields, the absence of direct contact of fuel and oxidizing gases at high temperatures mitigates the formation of pollutants like dioxins and nitrous oxides (NO_*x*_).^21–23^ The high-grade heat produced during the regeneration step can cater to the low-grade heat requirement in the reducer reactor, thus eliminating any external heat requirement.^23^ Moreover, advancements in efficient heating solutions offering near-unity conversion of renewable energy to heat negate the possible hindrance to the implementation of CL technologies requiring additional thermal input.^24^

**Fig. 1:**
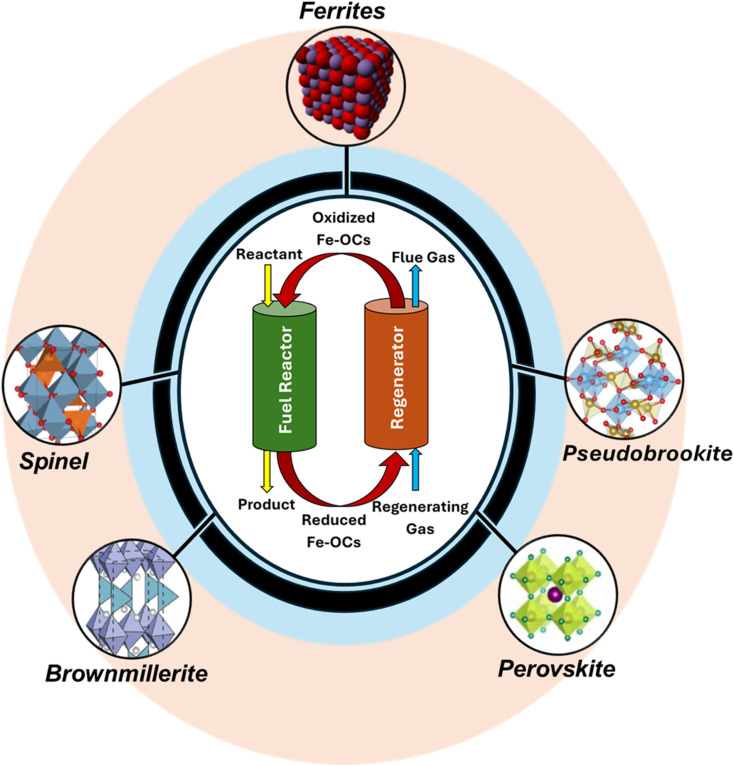
Prominence of metal ferrite derivatives in chemical looping. Brownmillerite structure adapted with permission from ref. 39. Copyright 2009 Springer Nature. Pseudobrookite structure adapted with permission from ref. 40. Copyright 2023 Elsevier. Perovskite structure adapted from a 2022 article published by MDPI (ref. 41) under a Creative Commons (CC BY) License. Spinel structure adapted from a 2020 article published by Springer Nature (ref. 42) under a Creative Commons Attribution 4.0 International (CC BY 4.0) License.

The choice of the metal oxide carrier plays a pivotal role in determining the technical feasibility and the economic viability of this technology.^5,25^ Transition metal oxides are highly favored for the CL process due to the high number of valence shell electrons, which facilitate ionic diffusion, providing a large number of active sites and correspondingly high reactivity.^26^ Several transition metal oxides, including but not limited to nickel oxide (NiO), copper oxide (CuO), iron oxide (Fe_2_O_3_), chromium oxide (Cr_2_O_3_), vanadium oxide (V_2_O_5_), manganese oxide (Mn_2_O_3_), cobalt oxide (Co_3_O_4_), ceria (CeO_2_), *etc.*, have been tested as probable candidates for the different CL schemes.^27–35^ Iron-based oxygen carriers (OC) have garnered increased attention due to their favorable thermodynamics towards many CL applications, environmentally friendly nature, abundance, and low cost.^28^ However, iron oxide faces several drawbacks, such as poor cyclic stability, poor kinetics, and high sintering.^30,36,37^ In order to eliminate the challenges associated with iron oxide carriers, additional active metal oxides, dopants, promoters, binders, and inert supports are incorporated, creating a class of OC that can be classified as ferrite-based oxygen carriers (Fe-OC).^38^ As illustrated in Fig. 1, the development of various Fe-OC composites with different crystal structures like spinel, brownmillerite, perovskite, and pseudobrookite has been of central importance to various CL applications.

Fe-OCs have demonstrated thermodynamic superiority, capacity to handle versatile feedstocks ranging from gases like methane (CH_4_) to solids like waste plastics, stable cyclic performance for an extended period of operation, and have proven to be suitable for several CL applications, including chemical looping combustion (CLC), gasification (CLG), partial oxidation (CLPO), reforming (CLR), and hydrogen generation (CLHG).^43–54^ The commercialization potential of the CL technology has been realized through successful demonstrations at bench-scale, sub-pilot scale, and pilot-scale testing using Fe-OCs. These developments highlight the multiscale approach for implementing Fe-OC for multiphase reaction engineering.^55^ While the superiority of Fe-OC has been established for a long time, a comprehensive review for understanding the role of each component incorporated in various ferrites is missing in the literature. This insight is crucial to achieving harmony between the chemical and physical properties of the OC and fine-tuning the OC composition for specific commercial applications. This review aims to understand the influence of different components on thermodynamics, the underlying mechanisms, the overall performance of ferrite OCs, and the feasibility of specific CL applications. This knowledge would be critical in tailoring and optimizing the OC composition, which is essential for the economic viability of the commercial process.

## 2 Fundamental insights into Fe-OC for performance enhancement

Understanding the fundamentals of ferrites as versatile OC is vital for their applications in CL systems. This section provides an in-depth exploration of the key fundamental aspects of Fe-OCs, focusing on how ionic diffusion influences its morphology, volume expansion, and structural integrity. It also examines the role of oxygen vacancies in enhancing lattice oxygen transport and overall reactivity. Additionally, the formation of core–shell structures and their advantages for improving stability and preventing sintering are discussed.

### 2.1. Role of ionic diffusion in Fe-OC

The morphology of Fe-OC is pivotal in influencing its surface area, reaction kinetics, and structural integrity, thereby directly affecting its overall performance. It is closely tied to ionic diffusion and changes in molar volume. The ionic diffusion during Fe-OC oxidation results in a net outward transport of Fe ions from the core to the surface, where they are oxidized to form Fe_2_O_3_. This results in an inward progressing Fe_2_O_3_ layer, leading to the complete oxidation of Fe-OC particle. Simultaneously, the oxidation reaction leads to volume expansion of Fe-OC, as observed from macroscopic studies on Fe–Ti OC by Li *et al.* and microscopic investigations on Fe_2_O_3_ OC by Cheng *et al.*^56,57^ The volume increases from 31.7 to 54.4 cm^3^ mol^−1^ in the case of FeTiO_3_ converting to Fe_2_TiO_5_ and from 7.1 to 30.5 cm^3^ mol^−1^ in the case of Fe converting to Fe_2_O_3_, both of which exhibit larger volume changes compared to many other transition metal oxides like NiO and CuO investigated in the literature.^57^ The high molar volumes of Fe-OC enhance the transport of Fe ions from the core to the surface and provide greater surface area for redox reactions, making Fe-OCs an attractive choice for CL applications.^58^ Therefore, understanding these ionic diffusion processes and the resulting changes in molar volume is essential for predicting and controlling the morphological transformations that occur during the reduction and oxidation cycles of Fe-OCs.

**Fig. 2:**
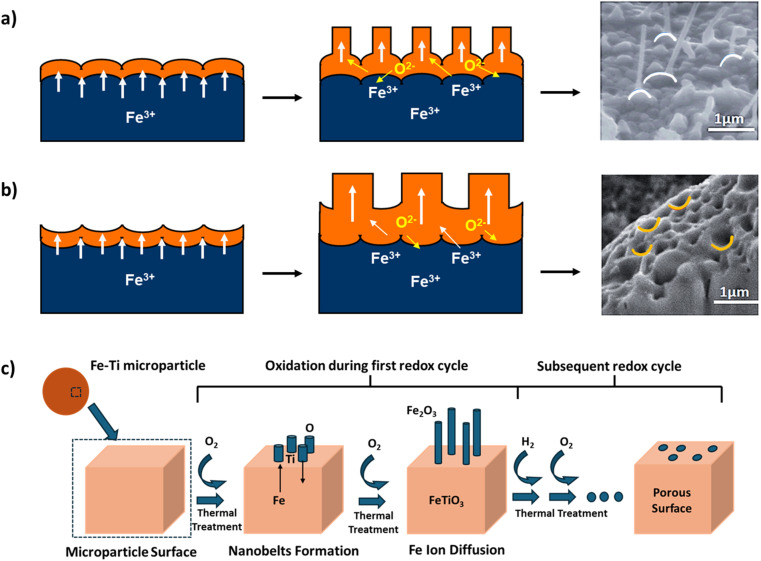
Nanoscale morphological transformations showing growth mechanisms for (a) nanowires, (b) nanopores of ferrite material, and (c) iron oxide nanobelt formation in Fe–Ti composite. White curves in (a) indicate a positive curvature, while yellow curves in part (b) highlight the negative curvature on the surface. (a) and (b) Adapted with permission from ref. 58. Copyright 2014 Royal Society of Chemistry. (c) Adapted with permission from ref. 59. Copyright 2015 Royal Society of Chemistry.

The performance and macroscopic behavior of metal oxides are heavily influenced by their nanoscale morphology, which is shaped by solid-state ionic diffusion. This morphology can vary significantly from single-metal systems to bimetallic ones. Lang *et al.* discuss the evolution of nanoscale morphology in such contexts. During the oxidation of Fe particles, the growth of nanostructures, such as nanowires and nanopores, depends significantly on the surface curvature of the grains. This process is governed by a stress-driven mass transport mechanism, where the curvature of Fe_2_O_3_ grains determines the type of nanostructure that forms. Positive surface curvature leads to the outward diffusion of Fe atoms, resulting in perpendicular nanowire growth, while negative surface curvature promotes oxide growth, leading to the formation of nanopores within the grain, as shown in Fig. 2(a) and (b), respectively.^58^ Lang *et al.* further extended the scope of their research to include bimetallic Fe–Ti particles. The difference in the diffusion rate of Fe ions compared to Ti ions resulted in the formation of nanobelts on the surface. Repeated redox cycles make these structures highly porous with improved morphological stability, as depicted in Fig. 2(c).^59^ These studies highlight the critical role of ionic diffusion in the design and performance of Fe-OCs.

### 2.2. Effect of oxygen vacancy in Fe-OCs

**Fig. 3:**
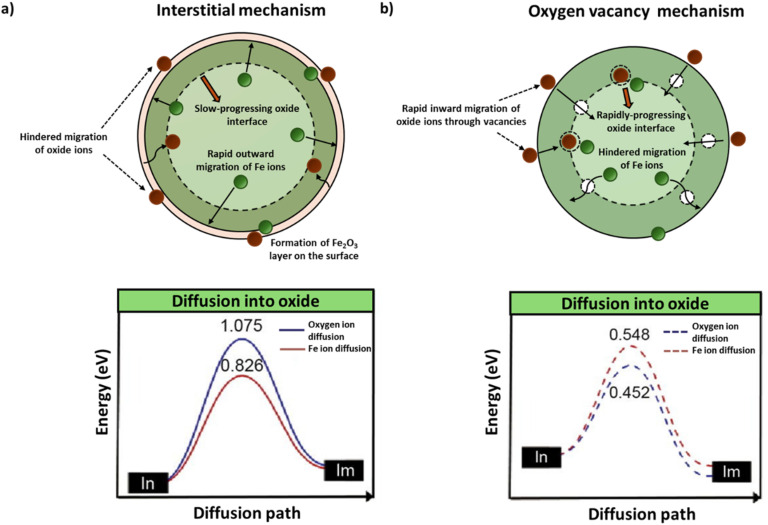
Ionic diffusion and corresponding activation energy diagram for Fe_2_O_3_ OC under (a) interstitial mechanism. (b) Vacancy mechanism. Green and white spheres indicate Fe ions and oxide ions, respectively, while white spheres depict oxygen vacancies. Energy diagrams adapted from a 2018 article published by Elsevier (ref. 57) under a CC BY-NC-ND 4.0 License.

During the reduction step of CL applications, the lattice oxygen from the Fe-OC, whether from the surface or bulk, is transferred to the hydrocarbon fuel, resulting in partial, selective, or complete oxidation. This process leads to phase transition and creates oxygen vacancies on the Fe-OC, which can significantly alter its morphology, chemical properties, and electronic structures. It also facilitates the transport of lattice O atoms from the bulk of the Fe-OC to the surface. Cheng *et al.* conducted DFT simulations to study the effect of oxygen vacancy on CH_4_ combustion using Fe_2_O_3_ as an OC. They stated that the presence of oxygen vacancies significantly increases the CH_4_ activation on the Fe-OC by lowering the dissociation barrier for the C–H bond of the CH_4_ molecule.^60^ During the oxidation reaction of Fe-OC in the absence of oxygen vacancies, the interstitial diffusion energy barrier for O moving inward is higher than the outward diffusion energy barrier of Fe, as shown in Fig. 3(a). This leads to a rapid formation of a Fe_2_O_3_ layer on the outer surface of the Fe-OC particle due to the reaction between outward diffusing Fe ions and oxidant. However, the high energy barrier for O diffusion decreases the rate of oxidation within the particle bulk. In the presence of oxygen vacancies, the inward diffusion barrier of O is reduced. It is slightly lower than the outward diffusion barrier for Fe, as observed from Fig. 3(b), thus facilitating the formation of iron oxide product at the Fe_2_O_3_–Fe interface. This results in a rapidly progressing Fe_2_O_3_ layer towards the center of the Fe-OC, enabling faster reaction rates for complete oxidation.^57^

Numerous atomistic studies on Fe-OCs have explored how dopants and support modifications can lower oxygen vacancy formation energy (Δ*E*_vac_) to enhance lattice oxygen transport and redox activity. Modifying Fe_2_O_3_ with TiO_2_ support decreases Δ*E*_vac_, enhancing the oxygen atom diffusion and substantially increasing the recyclability of the Fe–Ti composite OC.^60^ The addition of 5% Ni dopant to Ca_2_Fe_2_O_5_ lowers Δ*E*_vac_ by 58%, resulting in a substantial improvement of nearly 1149% in the Fe-OC's redox activity.^61^ In another study, it was observed that doping Fe_2_O_3_ with 1% Cu and 2% Co reduces Δ*E*_vac_ by 29% and 31%, respectively.^62,63^ Composite Fe-OCs have also been utilized in CLC applications. For example, adding CeO_2_ to Fe_2_O_3_ OC significantly reduces Δ*E*_vac_ from 9.56 eV (in pure Fe_2_O_3_) to 3.95 eV (in the CeO_2_/Fe_2_O_3_ composite OC). This facilitated the increase in the formation of surface oxygen vacancies, improving the composite material's performance compared to pure Fe_2_O_3_.^64^

### 2.3. Structural design for enhanced Fe-OC performance

The reactivity and stability of Fe-OCs can be significantly enhanced by modifying their structural design, with core–shell structures showing promise in CL applications. Core–shell structures often form during redox cycles through solid–solid reactions between the active material and an inert support. During these cycles, cations diffuse outward, transporting the active material from the core to the surface, forming a protective shell around the core. The sol–gel method is one of the major preparation techniques that enable the synthesis of core–shell OCs with controlled composition and structure, making them well-suited for CL applications.^65–67^

Core–shell structures often outperform homogeneous composite materials. Ma *et al.* found that Fe_2_O_3_@CeO_2_ core–shell OC exhibited significantly higher redox reactivity and stability compared to Fe_2_O_3_/CeO_2_ homogeneous composite Fe-OC in CLHG applications. This improvement was due to the core–shell structure's strong resistance to sintering, which prevents Fe cations from migrating to the particle surface, thus maintaining the OC's stability and performance. In contrast, the homogeneous composite OC suffers from significant sintering, with Fe cations accumulating on the surface, leading to reduced performance.^68^ The careful design of both core and shell materials is essential for superior CL performance. Blaschke *et al.* studied the impact of shell composition on the performance of OCs by using Fe_2_O_3_ as the core and preparing two samples with different shell compositions: yttrium-stabilized zirconium oxide with 8 mol% Y_2_O_3_ (YSZ8-CS) and yttria-stabilized zirconium oxide with over 10 mol% Y_2_O_3_ (YSZ10-CS). The ratio of weights of Fe_2_O_3_ and the support YSZ was kept constant. Both samples show increased pore volume during redox cycles, but YSZ10-CS develops poorly connected pores, reducing effective porosity and leading to sintering and agglomeration. In contrast, YSZ8-CS maintains a stable pore structure, avoiding “ink bottle” pores that trap gases, thereby preserving its effectiveness and overall performance.^69^ Yin *et al.* investigated Fe_2_O_3_ nanocores covered with a CeO_2_ shell. This configuration combined the superior ion conductivity of CeO_2_ with the high oxygen storage capacity of Fe_2_O_3_. The resulting Fe–Ce core–shell OC was highly influential in selectively providing oxygen from the CeO_2_ shell, which improved selectivity towards syngas production and enhanced CH_4_ conversion.^70^

## 3 Prominence of Fe-OCs in CL processes

Understanding the fundamental properties of the materials and their impact on the performance of the OC provides a strong foundation for exploring the Fe-OCs for various CL applications for carbon neutrality and clean energy production. The choice of active component greatly dominates the feasibility of the CL process. This section dwells on the thermodynamic feasibility, experimental evaluation, and scale-up studies of Fe-OCs for different CL applications.

### 3.1. Thermodynamic feasibility of oxygen release and uptake by Fe-OC

Fe-OCs have enabled the application of CL technology to produce a wide range of commodity chemicals. However, the thermodynamics of the Fe-OC during the oxidation and reduction reactions dictate the feasibility of the desired CL application. CLC, CLPO, and CLHG are the prominently pursued CL applications in the literature for achieving clean and sustainable energy production.

**Fig. 4:**
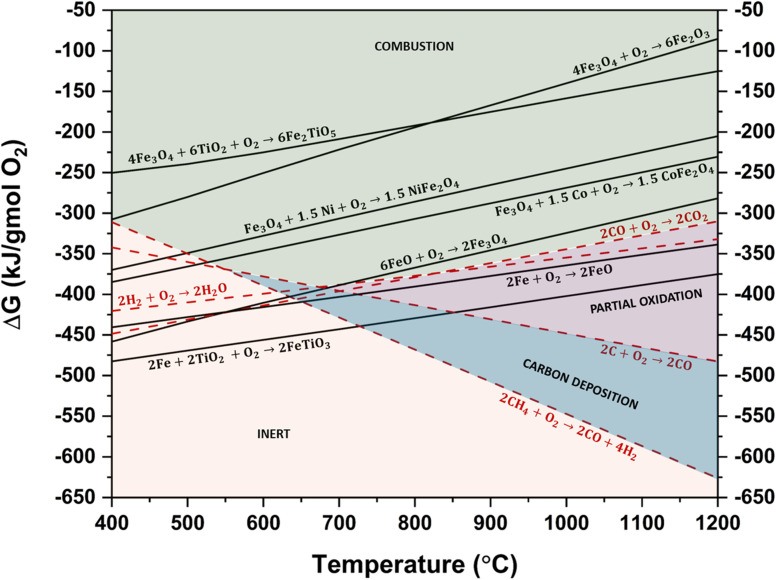
Ellingham diagram for various metal ferrite OCs.

Thermodynamic feasibility is influenced by several factors, including fuel conversion, CO_2_ purity, syngas purity, and product selectivity. In a commercial-scale CL process, oxygen carriers must favor complete thermodynamic fuel conversion while maintaining high selectivity toward the target product. Additionally, the kinetic activity of Fe-OCs should facilitate fuel conversion and product selectivity approaching thermodynamic limits. With the increasing emphasis on renewable feedstocks such as biomass, these properties become even more critical for achieving complete tar cracking and ensuring high product yields. The study of the Ellingham diagram presents a useful approach for determining the viability of the Fe-OC for a given CL process by evaluating the oxidation potential of the carrier. Fig. 4 illustrates the Ellingham diagram for some prominent Fe-OCs. The free energy of oxidation of the reduced counterparts of the Fe-OCs indicates the thermodynamic feasibility of Fe_2_TiO_5_, NiFe_2_O_4_, CoFe_2_O_4_, Fe_2_O_3_, and Fe_3_O_4_ for CLC application, while FeO and FeTiO_3_ can establish equilibrium with syngas during CLPO application.^43,71,72^ While this theoretical assessment indicates if a particular process is thermodynamically favored, an experimental examination is necessary to assess the kinetics and oxygen transport capacity (OTC) of the Fe-OC material. Therefore, the experimental evaluations are studied in Section 3.2 to investigate the practical application of Fe-OC for CL processes.

### 3.2. Experimental evaluation of Fe-OC for diverse CL processes

In order to enable complete carbon capture during the CLC process, it is essential to utilize materials with high oxygen transport capacity, similar to Fe_2_O_3_, which has a theoretical OTC of 30.06%. As observed in Section 3.1, Fe-OCs, such as CoFe_2_O_4_, exhibit a high oxidation potential and demonstrate a high theoretical OTC of 27.28%. With the OTC being similar to Fe_2_O_3_ and thermodynamics favoring complete combustion, CoFe_2_O_4_ has been extensively examined for CLC application. Evaluation conducted by Fan and co-workers to synthesize OC with high OTC using Cu, Fe, and Mn as constituent metals revealed the notable performance of CuFeMnO_4_ Fe-OC for CLC, owing to the theoretical OTC of 20.04%, synergistic effect of the trimetallic system, and distinct reaction profile.^73,74^ Composite OC like NiFe_2_O_4_ also exhibits a high theoretical OTC of 27.31%, allowing complete carbon capture and improved tar cracking properties arising from the high catalytic activity of the metallic Ni formed in the reduced phase and unique spinel structure.^72,75^

As opposed to CLC, where a higher OTC is favored, materials with a lower oxidation potential and lower OTC than Fe_2_O_3_ are promising for CLPO. Ca_2_Fe_2_O_5_, having a low theoretical OTC of 17.66%, has revealed high conversions and remarkably high syngas selectivity, as showcased by Shah *et al.*^61^ More than 99% CH_4_ conversion with >98% syngas selectivity obtained can be ascribed to the brownmillerite type structure and favorable thermodynamics of oxygen transfer for partial oxidation. Moreover, the ability of Ca_2_Fe_2_O_5_ to regenerate using CO_2_/H_2_O increases the overall syngas yield.^76^ Other probable candidates for CLPO applications, such as Li_5_FeO_4_, SrFe_12_O_19_, *etc.*, with a maximum OTC of 12.13% and 23.73%, respectively, possess bifunctional properties with both catalytic and oxygen transfer properties.^77,78^ The Fe–Ti-based synthetic OC developed by Ohio State University (OSU) has shown excellent syngas yield due to its oxygen migration control, achieving syngas purities as high as 90%.^43,47^ While theoretical OTC helps identify the potential CL application for Fe-OC, it is important to note that the thermodynamics and kinetic activity of the Fe-OC play a crucial role in determining actual OTC and the product selectivity, as seen in the case of SrFe_12_O_19_.

CLHG processes are commonly combined with CLC or CLPO, where the oxidizer produces H_2_ by steam oxidation of the OC. The favorable thermodynamics of OC for steam regeneration are an essential prerequisite for its use in CLHG. The H_2_ yield from the material highly depends on the degree of reduction of Fe-OC, which is higher in CLC than in CLPO. Thus, to maximize the yield of H_2_, CLC followed by the CLHG process was studied using metal ferrites like CaFe_2_O_4_, Co_0.85_Fe_2.15_O_3_, NiLa_0.2_Fe_1.8_O_4_, *etc.*, which display higher redox stability over Fe_2_O_3_.^79–82^ At the same time, the ability of Ca_2_Fe_2_O_5_ to regenerate completely using steam has made it a promising candidate for syngas-H_2_ co-generation.

**Fig. 5:**
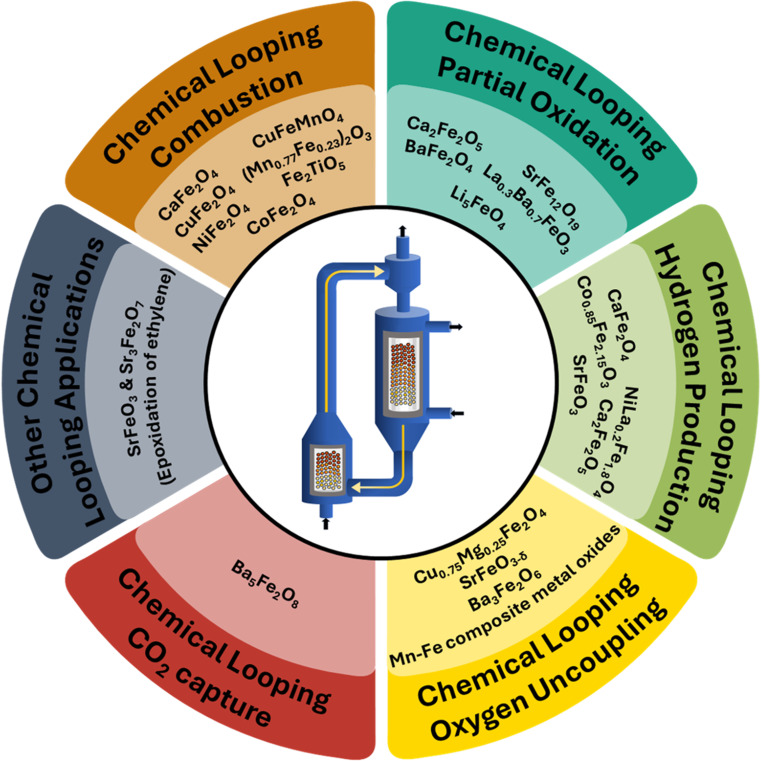
Application of ferrites in different CL processes. The central CL unit adapted from a 2024 article published by Elsevier (ref. 20) under a Creative Commons Attribution 4.0 International (CC BY 4.0) License.

Apart from the conventional applications of the CL processes, several OCs have been developed for applications such as CLOU, oxidative dehydrogenation, preferential oxidation, selective dehydrogenation, *etc.*, as summarized in Fig. 5. SrFeO_3_ and Sr_3_Fe_2_O_7_ in a 1 : 1 ratio have been utilized for chemical looping ethylene epoxidation and have shown significantly higher yields of ethylene oxide as compared to conventional processes.^83–85^ Controlled oxidation enables a four times higher yield than conventional processes due to its property of controlled oxidation. Similarly, a mixture of Ba_5_Fe_2_O_8_ and Ba_3_Fe_2_O_6_ can be utilized for CLOU with inherent CO_2_ capture at high temperatures, leveraging the superior capture performance of the former and higher oxygen uncoupling of the latter. Table 1 summarizes details on the structure, method of preparation, and applications of some Fe-OCs.

**Table 1:** Classification of Fe-OC based on phases and the effects observed on CL processes due to different preparation methods and experimental conditions

OC	Preparation method	Experimental temperature	Application	Key findings	Ref.
**Spinel**					
NiFe_2_O_4_	Sol–gel	800 °C	CLHG	Grain coalescence and surface densification lead to deactivation, which can be prevented by incorporating Al	86
Cu_*x*_Mg_1−*x*_Fe_2_O_4_	Mechanical mixing and calcination	900–1000 °C	CLC	∼98.9% conversion of waste biomass	84
				The addition of Mg improved crushing strength above 157.19 MPa	
M_*x*_Fe_3−*x*_O_4_, M = Cr and Mn	Iron ore	850 °C	CLC	Fe–Cr-based OC demonstrates improved stability and performance due to the spinel structure	87
Cu_*x*_Mn_1−*x*_Fe_2_O_4_	Co-precipitation	600–700 °C	CL-SMR	Cu_0.6_Mn_0.4_Fe_2_O_4_ enabled CH_4_ conversion of ∼98.7% and H_2_ yield of ∼81.02%	88
Cu_0.2_Co_0.8_Fe_2_O_4_	Sol–gel	<750 °C	CL-CO_2_ splitting	Improved reduction facilitated by Cu and Co as co-dopants	89
				Increasing Cu doping results in the formation of impurities detrimental to the CO_2_-splitting process	

**Perovskite**					
La_0.75_Sr_0.25_FeO_3_-encapsulated Co_3_O_4_–NiO	Modified Pechini	600 °C	CL-RWGS	Promotes oxygen defects in the metal oxide core during H_2_–CO_2_ redox cycles, enabling higher yield of CO	90
La_0.5_Sr_0.5_Fe_0.5_Co_0.5_O_3−*δ*_	Glycine-nitrate combustion and spray drying	900 °C	CLC	La substitution increases the OTC	91
				The selectivity towards CO/H_2_ and the reactivity with CH_4_ was reduced	
La_1−*x*_Sr_*x*_FeO_3_	Sol–gel	1000 °C	CLR	Partial substitution of La^3+^ by Sr^2+^ causes electronic imbalance, which is compensated by oxidation of a fraction of Fe^3+^ to Fe^4+^ and/or generation of oxygen vacancies	92
				The optimum range of the degree of Sr substitution is *x* = 0.3–0.5	
LaFe_1−*x*_Co_*x*_O_3_	Combustion	850 °C	CLR	The surface adsorbed oxygen essential for complete oxidation of CH_4_ increases with Co substitution	93
				The optimum degree of Co substitution is *x* = 0.3	
Sr(Fe_1−*x*_Cu_*x*_)O_3−*δ*_	Calcination method	600–800 °C	CLC	Can sustain very high temperatures (>1280 °C) with a crushing strength >39.32 MPa	94

**Pseudobrookite**					
Fe_2_TiO_5_	Mechanical mixing followed by pulverization	875–925 °C	CLC	Improved diffusion of CH_4_ through the Fe-OC led to an enhanced activity of Fe_2_TiO_5_ supported on Al_2_O_3_	95

**Brownmillerite**					
Ca_2_Ni_0.25_Fe_1.75_O_5_	Sol–gel	900 °C	CL-SR	Ni induces structural changes to the brownmillerite lattice, increasing the distortion of the FeO_6_ octahedra. The structural changes lead to improved lattice oxygen activity	96
Ca_2_Fe_2_O_5_	Sol–gel	850 °C	CL-SR	Reduction of the migration energy barrier of lattice oxygen between bulk and surface improved the reactivity	97
MgO supported Ca_2_Fe_2_O_5_	Solid state synthesis	1000 °C	CLR	CO_2_ counter-oxidation enabled ∼20% higher CO_2_ utilization compared to tri-reforming	98

### 3.3. Performance evaluation of Fe-OC for scaled-up CL applications

With a realization of the scale-up potential of various Fe-OCs, various demonstrations on bench-scale, sub-pilot scale, and pilot scale have been conducted, highlighting the capability of chemical looping technology for clean energy production and achieving carbon neutrality. This section draws attention to the scale-up attempts of CL spanning from kW_th_ to MW_th_ scale using synthetic and natural Fe-OCs for diverse feedstocks.^99^

Southeast University has experimentally investigated CLC in continuous fluidized bed reactors ranging from a scale of 1–50 kW_th_ for a wide range of solid fuels using synthetic Fe-OC.^100–103^ These tests proved the potential of Fe-OC as a low-cost carrier for commercial coal-fueled CLC units. OSU demonstrated a 25 kW_th_ followed by a 250 kW_th_ high-pressure moving bed reactor configuration for syngas utilization using Fe–Ti OC to enable H_2_ production with 100% CO_2_ capture.^46^ Another 250 kW_th_ demonstration highlighted the supremacy of Fe–Ti OC for near 100% conversion of direct solid fuels.^48^ Over 600 hours of testing using Fe-OC for biomass gasification in a 25 kW_th_ sub-pilot unit exhibited excellent tar-cracking capabilities, high attrition resistance, and stable reactivity of the Fe–Ti OC for extended redox operation.^44^

Incorporating additional metal oxides, such as CuO and NiO, into Fe-OCs has demonstrated the potential to enhance their reactivity while maintaining cyclic stability, positioning these OCs as promising candidates for the scale-up of CL technology.^104^ In a 10 kW_th_ demonstration for biomass gasification, Fe–Ni bimetallic OCs improved the gasification efficiency compared to single metal OC owing to the catalytic activity of Ni.^105,106^ Investigations conducted in a 10 kW_th_ and 50 kW_th_ CLC unit using coke oven gas and CH_4_, respectively, established the superiority of supported Cu–Fe OC for extended redox testing and for the achievement of autothermal operation.^107–109^

Besides synthetic Fe-OC, natural ores have gained attention for CL applications owing to their low cost and excellent sintering resistance. Experimental evaluation in 10 kW_th_ and 100 kW_th_ by Chalmers University using ilmenite and a mixture of ilmenite–hematite demonstrated high fuel conversion for low-volatile solids with a projected Fe-OC lifespan of 700–800 hours.^110–115^ Natural mineral-based OCs, like hematite, have also been evaluated in fluidized bed CLC reactors.^116^ 25 kW_th_ scale CLG investigation for coal and biomass achieved high syngas purity with high carbon conversion.^117^ A CLC plant on a 1 MW_th_ scale at Technische Universität Darmstadt demonstrated autothermal operation for coal CLC using both ilmenite and hematite.^118–120^ These commercial-scale demonstrations show that natural iron ores could be a suitable material with good mechanical properties, low attrition, good reactivity, high OTC, and low cost. Table 2 provides a further overview of some of the scale-up attempts for CL technology reported in the literature.

**Table 2:** A non-exhaustive list of scale-up attempts for CL processes with Fe-OC

Fe-OC	Feedstock	Hours of operation	Scale	Reactor configuration	Ref.
**Synthetic iron-based**					
Fe_2_O_3_-supported	Coal, biomass	200–600	25–250 kW_th_	Moving bed	44, 48 and 121
Fe_2_O_3_ and Fe_2_O_3_/Al_2_O_3_	Biomass	30, 60	10 kW_th_	Fluidized bed	105 and 122
Fe–Ni and Fe–Cu on Al- and MgAl-based supports	Natural gas, biomass, coke oven gas	2–40	10–50 kW_th_	Fluidized bed	106, 107, 123 and 124

**Natural ore based**					
Iron ore	Biomass and coal	26	100 kW_th_	Fluidized bed	125
Ilmenite	Coal, petcoke, biomass, CH_4_, CO, and H_2_	22–160	10 kW_th_–12 MW_th_	Fluidized bed	113–115 and 126–129
Ilmenite–iron ore	Coal and biomass	110	1 MW_th_	Fluidized bed	119
Hematite	Coal	100	5 kW_th_	Fluidized bed	116
Ilmenite–manganese ore	Biomass and petcoke	18	100 kW_th_	Fluidized bed	130

## 4 Challenges and opportunities

In order to implement the potential benefits of the CL systems on a commercial scale, the ability of the Fe-OC to maintain reactivity and structural stability over an extended period of testing is crucial. This section highlights some of the key challenges encountered in the use of Fe-OCs for CL applications. Moreover, detailed insight into plausible composition modifications, which can ameliorate the barriers to the implementation of Fe-OC on a commercial scale, has been discussed.

### 4.1. Cyclic stability and active material dispersion of Fe-OC

Fe-OCs face a significant challenge in maintaining cyclic stability during the redox process owing to poor mechanical strength and active site dispersion on the OC, which can be improved by the incorporation of inert support material. Common supports that have demonstrated an improvement in the material stability for extended redox cycles include but are not limited to Al_2_O_3_, TiO_2_, ZrO_2_, YSZ, CaO, MgO, and CeO_2_. A reactivity improvement in the order of Al_2_O_3_ > SiO_2_ > TiO_2_ > unsupported > CaO is observed in Fe_2_O_3_, which could be attributed to the enhanced gas diffusion through the interconnected pores in Al_2_O_3_, SiO_2_, and TiO_2_-supported OCs. Moreover, Al_2_O_3_ and TiO_2_ also contribute to the increase in mechanical strength of the Fe-OC during the fluidized bed testing.^131^ Tijani *et al.* found that the use of ZrO_2_ and CeO_2_ supports significantly decrease the activation energy barrier by ∼4 times during the oxidation step compared to Al_2_O_3_ and TiO_2_.^132^ In order to prevent the eventual loss of reactivity by using CeO_2_ support, the Fe–Ce structure was stabilized by adding ∼7 wt% of ZrO_2_ in the fluorite configuration of CeO_2_. This leads to the formation of oxygen vacancies and mitigates the formation of CeFeO_3_, increasing the redox stability of the Fe-OC.^133^

The active material dispersion and reactivity can be further enhanced using mesoporous inert supports like SBA-15 to synthesize nano-scaled carriers. Kumar *et al.* observed a remarkable 1000% improvement in reactivity using LaFeO_3_-SBA-15 compared to the bulk carrier for CLC application owing to the formation of a reactive cubic phase at the nanoscale.^20^ Fe_2_O_3_ nanoparticles synthesized by Liu and co-workers using the SBA-15 matrix for CLPO application display >99.3% selectivity toward CO formation from CH_4_ and a stable performance for 75 redox cycles. The 70% improvement in conversion rate and ∼200% higher CO concentration compared to the bulk carrier can be attributed to the lower adsorption and dissociation energies of CH_4_ in the presence of nanoparticles.^134^

In addition to the choice of inert support, the synthesis technique used has a profound effect on the performance of Fe-OC in chemical looping. Mechanical mixing, freeze granulation, dry and wet impregnation, co-precipitation, and sol–gel are commonly practiced methods to prepare Fe-OCs. Fe_2_O_3_–Al_2_O_3_ OCs prepared by the sol–gel process in the Fe : Al_2_O_3_ molar ratio of 2 : 1 demonstrate better reactivity and stable performance across multiple redox cycles, among other preparation methods like mechanical mixing and dipping.^135^ This can be attributed to the excellent homogeneity and control over microstructure in the sol–gel method, which leads to the formation of Al_3_Fe_5_O_12_.^28^ Another study identified that the conventional wet impregnation method for preparing Fe-OCs is challenged by poor dispersion of active material on the support and insufficient penetration into the binder material. However, this challenge was resolved using the ultrasonic wet impregnation technique, characterized by a reduction in the crystalline size and better dispersion of the active material, enabling higher conversions of CH_4_.^136^

### 4.2. Loss of reactivity and performance degradation

The incorporation of inert support enhances the cyclic stability of the material. However, a decrease in the performance of the Fe-OC has been observed with increasing redox cycles, arising due to degradation in the OTC.^137^ Dopant modification is an effective technique to retain the reactive capability of the Fe-OC over an extended number of cycles. Alkaline metals are commonly studied as dopants since they act as an electron donor and weaken the Fe–O bond. Liu *et al.* observed that 2.6 mol% K doped Fe–Al carrier demonstrates superior CO_2_ selectivity and stable reactivity for ∼50 redox cycles. This can be ascribed to the stiffening of the Al_2_O_3_ matrix in the presence of K, providing a stable path for [O] migration.^137^ Similarly, adding 5% Na using sodium salt, like NaCl, as a dopant in the Fe–Al matrix also helps increase the yield of CO_2_ in CLC.^138^ Isovalent metal oxide dopants like 1% La dramatically enhance the reactivity for the combustion of CO and regeneration by ∼233% and 266%, respectively. This can be attributed to the lowering of the energy barrier due to La for CO bond activation.^139^ Doping 2% La on Fe–CeO_2_-based OC results in the Fe–La_0.02_Ce_0.95_O_1.99_ phase, facilitating a very high oxygen transport by altering the thermodynamic stability of CeO_2_. Increasing the dopant concentration beyond 10% does not contribute significantly to further enhancement owing to La phase separation to form LaFeO_3_ with poor reactivity. The cyclic stability testing with 10% La doping on the CeO_2_ matrix results in the formation of Fe–La_0.1_Ce_0.9_O_1.95_. La-doped Fe-OC demonstrated excellent recyclability over 50 redox cycles, whereas the undoped Fe-OC lost the ability to regenerate after 11 cycles.^140^

### 4.3. Carbon and ash deposition

Carbon deposition can significantly compromise the Fe-OC stability and purity of the products, which may negate the inherent product separation advantage of CL systems. Carbon can exist in amorphous, filamentary, and crystalline graphitic forms and get deposited on the Fe-OC particles.^141,142^ Deeply reduced Fe-OCs are susceptible to carbon deposition owing to the difficulty in lattice oxygen migration through the metallic Fe and FeO phases.^143,144^ The two major strategies to prevent carbon deposition are (1) dopant and support modification to mitigate the C–H bond cleavage. (2) Synthesis of composite metal oxides to increase the lattice oxygen mobility for enhancing the oxidation of deposited carbon.

**Fig. 6:**
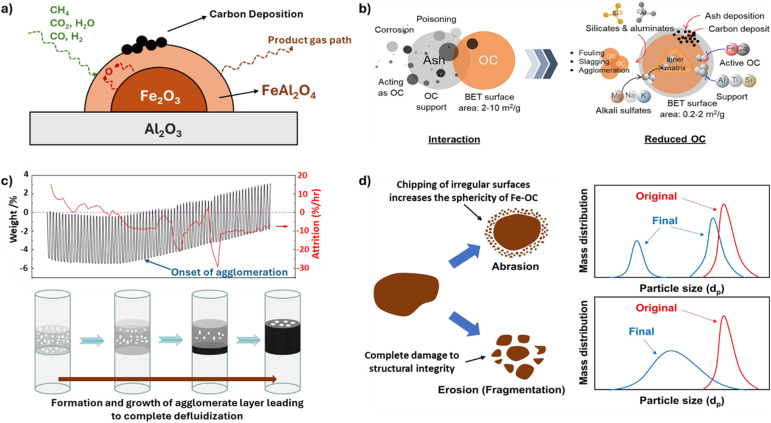
Challenges encountered in CL applications while using Fe-OC. (a) Onset of carbon deposition. Adapted with permission from ref. 149. Copyright 2023 Elsevier. (b) Ash deposition. Adapted from article (ref. 150) published by Elsevier under a Creative Commons Attribution 4.0 International (CC BY 4.0) License. (c) Formation and growth of agglomerate layer. Adapted with permission from ref. 151. Copyright 2023 Elsevier. (d) Fe-OC attrition. Adapted with permission from ref. 152 and 153. Copyright 2016 and 2023 Elsevier.

Modifying the natural iron ore-based OC by adding alkali metal, Ni, and Cu-based dopants blocks the active sites for carbon accumulation and increases the mobility of lattice oxygen by alleviating the iron–oxygen interaction. MgO modification, 10% KNO_3_ decoration, and co-doping of K and Cu can improve the ability to remove carbon by enhancing oxygen mobility for natural Fe-OCs.^145–148^ Synthetic Fe–Al OCs face the challenge of carbon deposition owing to the increase in acidic sites, which enhances C–H bond cleavage and the formation of FeAl_2_O_4_ spinel with low [O] mobility, as shown in Fig. 6(a).^154,155^ K_2_CO_3_-doped Fe–Al-based OCs have demonstrated the prevention of carbon deposition by limiting the concentration of acidic sites. Y-Doped Fe–Al OCs sintered at 1200 °C form a garnet structure Y_3_Fe_*x*_Al_5−*x*_O_12_ (*x* = 1, 2, 3), demonstrating high selectivity for syngas production by avoiding carbon deposition.^156,157^ Adding MgO in Fe–Al OCs gives rise to the phase of MgAl_2_O_4_, which mitigates the interaction between iron oxide and Al_2_O_3_, reducing carbon deposition.^158^ CeO_2_-Modified Al_2_O_3_ support, or Fe_2_O_3_ supported on 40% CeO_2_, leads to the formation of a perovskite CeFeO_3_ structure that can generate channels for [O] migration, eliminating carbon accumulation on OC.^52,68,159–161^ Besides dopant and support modification, iron-based composites are known to enhance the reactivity and alleviate carbon deposition. Spinel structures like CuFe_2_O_4_, BaFe_2_O_4_, 4% Cu–4% Ce doped CoFe_2_O_4_, and 70% CeO_2_–NiFe_2_O_4_ have demonstrated microstructural integrity over multiple redox cycles and facilitated higher [O] release to prevent carbon deposition.^104,162–168^ Perovskite structures like La_0.5_Ce_0.5_FeO_3_, BaFe_0.4_Sn_0.6_O_3−*δ*_, and LaSrFe_2−*x*_Co_*x*_O_6_ improve the reactivity and mitigate carbon deposition owing to increased oxygen migration due to structural distortion.^169^ Hexaaluminates like BaFe_*x*_Al_12−*x*_O_19_ and LaFe_3_Al_9_O_19_ have proved to be promising candidates for OC with low coking and high reactivity.^170,171^

Solid feedstocks, including fossil fuels like coal, sustainable carbon sources like biomass, and non-conventional feedstocks like municipal waste, present a challenge of ash deposition, as illustrated in Fig. 6(b). The components in ash include SiO_2_, Al_2_O_3_, Fe_2_O_3_, CaO, TiO_2_, K_2_O, MgO, Na_2_O, and P_2_O_5_. While the effects of ash deposition are not completely adverse, they can cause physical damage to the Fe-OC particles by clogging the pores, resulting in loss of reactivity and inducing mechanical stresses, leading to crack formation in the particles.^172,173^ Chemical interactions between fuel and low melting components like Ca and K from ash can lead to agglomeration of the particles and corrosion of the CL equipment.^150,174^ While ash majorly has detrimental effects on the Fe-OC, some active components present in the ash improve the reaction rate. Ca-based oxides in the ash prevent the formation of side products like Al- and Si- complexes, which are responsible for deactivating the Fe-OCs. The oxides of transition metals in the ash enhance the redox stability, and components like Fe_2_O_3_ and CaSO_4_ promote the kinetic activity of the oxygen carrier.^175^

In order to tackle the deteriorating effects of the ash, some techniques like washing for removing soluble salts of K, Ca, Si, P, Cl, Mg, and Na and pickling to remove water-insoluble salts by acid treatment are well-known.^176,177^ In addition to biomass pre-treatment, structural and compositional optimization to prevent the blocking of pores and improve redox activity can be beneficial in negating inhibitory effects like agglomeration and deactivation arising from ash deposition. NiFe_2_O_4_, MnFe_2_O_4_, CoFeAlO_*x*_, CuFe_2_O_4_, Ca_2_Fe_1.8_Co_0.2_O_5_ are found to maintain excellent redox activity and mitigate agglomeration, which can subside the adverse effects observed due to ash deposition.^178–180^

### 4.4. Durability of Fe-OC particles over long-term redox cycling

Due to the low Tammann temperatures (*T*_Tam_) of iron oxides, Fe-OCs tend to agglomerate, causing the deactivation of the OC and a decrease in oxygen diffusion. In moving and fluidized bed CL technologies, particle agglomeration can lead to reactor bridging and defluidization, which can hinder the long-term cyclic performance of the material, as shown in Fig. 6(c).

Some bimetallic Fe-OCs like CoFe_2_O_4_, Fe_2_TiO_5_, and Ca_2_Fe_2_O_5_ have high melting points of ∼1570 °C, ∼1550 °C, and ∼1450 °C, respectively correspondingly increasing *T*_Tam_.^181,182^ This helps the Fe-OCs to sustain higher operational temperatures, which can be beneficial for improving activity and thermodynamic conversions. Also, adding inert support ameliorates sintering as it increases the heat capacity of the material, thus raising the *T*_Tam_ of the Fe-OC. Cho *et al.* reported a stable performance of Fe_2_O_3_–Al_2_O_3_ (2 : 3) particles for 40 cycles without defluidization due to agglomeration.^183^ A bimetallic NiFe_2_O_4_ carrier supported on a SiO_2_ alleviates sintering compared to unsupported NiFe_2_O_4_. However, after 15 redox cycles, Ni–Fe carriers demonstrate a block structure due to agglomeration, causing a decrease in reactivity.^184,185^ Therefore, nanocomposites of NiFe_2_O_4_ impregnated on silica matrix were synthesized, which prevents particle aggregation due to enhanced dispersion of spinel NiFe_2_O_4_. Olhero and co-workers conducted a study to understand the effect of heat treatment on agglomeration using zinc-substituted Ni ferrite (Ni_0.8_Zn_0.2_Fe_2_O_4_) powders. Increasing the calcination temperature from 400 °C to 1200 °C causes a 776% increase in the average particle size and a 99.9% decrease in the specific surface area due to agglomeration.^186^ Scheffe *et al.* synthesized Co_0.85_Fe_2.15_O_4_ Fe-OC *via* atomic layer deposition on ZrO_2_, demonstrating stable and improved reactivity and ameliorating agglomeration.^80^

The commercial CL process would involve Fe-OC fluidization for thousands of hours under varying temperatures and the constant swing of oxidation states.^187^ The continuous mechanical, thermal, and chemical stresses can lead to chipping, disintegration, abrasion, or fragmentation of the Fe-OC, broadly classified as attrition.^188^ As illustrated in Fig. 6(d), abrasion, characterized by the chipping of uneven surfaces of the parent Fe-OC, is majorly observed at lower particle circulation rates. Fragmentation, observed at higher Fe-OC circulation rates, leads to many sub-particles forming fragments. Attrition of Fe-OCs results in particle make-up cost, which is a function of attrition rate (*A*), solid circulation rate, and cost of Fe-OC manufacture. Therefore, attrition can greatly influence the overall operating costs of the CL plant, and hence, tremendous efforts are focused on decreasing the attrition rate and increasing the Fe-OC lifetime. Adanez and co-workers found that 20 wt% Fe_2_O_3_ supported on Al_2_O_3_ demonstrates low *A* of 0.09–0.14% per h, translating into a Fe-OC lifetime of 700–1150 hours at an operating temperature of ∼900 °C.^189^ Pans *et al.* also utilized low-cost iron ore containing 76 wt% Fe_2_O_3_ in the CLC unit to generate syngas at 830–930 °C and observed a low *A* of 0.05% per h, corresponding to a lifetime of 2000 hours.^190^ Mei *et al.* utilized four different Fe-OC compositions: 65.6% Mn_3_O_4_–18.6% Fe_2_O_3_, 67.5% Mn_3_O_4_–8.4% Fe_2_O_3_, 71.8% Mn_3_O_4_–6% Fe_2_O_3_, 80.7% Mn_3_O_4_–5.2% Fe_2_O_3_, dispersed over Al_2_O_3_ as the inert support and observed that Fe-OC composed of 65.6% Mn_3_O_4_ achieves the lowest *A* of 0.0625% per h increasing the lifetime to 1600 hours. The remaining compositions had an attrition rate in the range of 0.1–0.67% per h with an OC lifetime of 1000–150 hours.^191^ Chung *et al.* have demonstrated stable performance of Fe-OC supported on Al-based inert for over 3000 cycles.^192^ It can be concluded that Al-based supports have proven to be promising in enhancing the lifetime of Fe-OC. Moreover, it has been observed for most of the synthetic Fe-OCs that the crushing strength increases with an increase in calcination temperature from 950–1600 °C but leads to a loss in the reactive capability of the material. Natural ores of iron-containing mixed-metal oxides of SiO_2_, Al_2_O_3_, CaO, Na_2_O, K_2_O, MgO, and TiO_2_ show a positive effect on reducing the A. However, fewer research efforts have focused on natural Fe-OC due to their low reactivity.^153^ While attrition presents a significant challenge to CL technology, the techno-economic evaluation conducted by Zhang *et al.* estimates that even with frequent solid make-up, the Fe-OC based CL process can be economically more attractive compared to the current state-of-the-art processes for commodity chemical production.^50^

Moreover, it is important to understand that the target application and the method of operation critically influence the choice of the active material and the Fe-OC composition. While the addition of a component may increase the attrition rate of the Fe-OC particles, it may prevent the deactivation of the Fe-OC due to carbon deposition. If the operational strategy of the process involves using fluidized beds, the addition of the component might be impractical since the process would require frequent solid makeup due to low OC lifetime. However, the Fe-OC can be well-suited for a fixed bed process where strength can be compromised to prevent carbon deposition for extended periods of operation. Similarly, increasing the strength and lowering the attrition rates may result in a compromise with the reactivity of the Fe-OC. In practice, the OC developed for larger scales of operation involves a delicate balance and an optimized trade-off of the extent to which each challenge is tackled by incorporating multiple components. Therefore, the precise compositions of most of the Fe-OCs that have been tested for a higher scale of operation are protected in the form of proprietary information.

## 5 Conclusion and perspectives

Chemical looping emerges as a highly promising technology in the pursuit of sustainable energy solutions. Metal ferrites have shown great potential as OCs for CL processes, offering a path toward reducing greenhouse gas emissions and improving the sustainability of industrial chemical production. Nevertheless, there remains a significant need to refine these materials and their associated processes to address current limitations and challenges. This review has thoroughly explored metal ferrites, highlighting their fundamental properties and critical role in advancing the efficacy of chemical looping processes. Innovation in Fe-OC is geared towards boosting OTC, increasing surface reactivity, and enhancing thermal stability. These improvements can be achieved through the development of composite metal ferrite and bimetallic systems. Employing nanoscale engineering and surface modification techniques could also markedly enhance the overall performance of Fe-OC for novel CL processes. Additionally, process optimization is vital for refining ferrite-based CL processes. This involves using sophisticated computational models and simulations to refine reactor designs and operational parameters, ensuring optimal interaction between metal ferrites and reactants and reducing energy losses. Integrating robust systems that align CL with current industrial practices could ease the adoption and implementation across various sectors. As these technologies have completed successful pilot testing and are progressing toward commercialization, enhancing lifecycle assessments to meet sustainability criteria will also be crucial. Fe-OCs-based CL processes can offer cost advantages over other transition metal oxide-based CL technologies for producing commodity chemicals. Therefore, the ongoing development of Fe-OCs and their derivative CL processes is expected to continue playing a critical role in advancing sustainable technologies, which will significantly contribute to scientific efforts and industrial practices to mitigate climate change and enhance clean energy production.

## Abbreviations

BTU: British thermal units
H_2_: Hydrogen
CH_3_OH: Methanol
C_2_H_5_OH: Ethanol
NH_3_: Ammonia
ATR: Auto-thermal reforming
SMR: Steam methane reforming
CL: Chemical looping
CO_2_: Carbon dioxide
H_2_O: Steam
CO: Carbon monoxide
NO_*x*_: Nitrous oxides (NO_*x*_)
NiO: Nickel oxide
CuO: Copper oxide
Fe_2_O_3_: Iron oxide
Cr_2_O_3_: Chromium oxide
V_2_O_5_: Vanadium oxide
Mn_2_O_3_: Manganese oxide
Co_3_O_4_: Cobalt oxide
CeO_2_: Ceria
OC: Oxygen carriers
Fe-OC: Ferrite-based oxygen carriers
FeTiO_3_: Iron-titanium oxide
CH_4_: Methane
CLC: Chemical looping combustion
CLG: Chemical looping gasification
CLPO: Chemical looping partial oxidation
CLR: Chemical looping reforming
CLHG: Chemical looping hydrogen generation;
Δ*E*_vac_: Vacancy formation energy
YSZ8-CS: 8 mol% yttria-stabilized zirconium oxide core–shell
YSZ10-CS: 10 mol% yttria-stabilized zirconium oxide core–shell
OTC: Oxygen transport capacity
OSU: Ohio State University
Na: Sodium
MgO: Magnesium oxide
K_2_CO_3_: Potassium carbonate
*T*_Tam_
: Tammann temperatures
Ni_0.8_Zn_0.2_Fe_2_O_4_: Zinc-substituted Ni ferrite
*A*
: Attrition rate

## Author contributions

Tanay A. Jawdekar and Ishani Karki Kudva contributed equally to this work and are co-first authors. Tanay A. Jawdekar: conceptualization, formal analysis, methodology, visualization, writing – original draft, writing – review & editing. Ishani Karki Kudva: conceptualization, methodology, visualization, writing – original draft, writing – review & editing. Sudeshna Gun: writing – original draft, visualization, writing – review & editing. Shekhar G. Shinde: writing – original draft, visualization, writing – review & editing. Ashin A. Sunny: writing – original draft, visualization, writing – review & editing. Zhuo Cheng: supervision, writing – original draft, writing – review & editing. Liang-Shih Fan: resources, supervision, writing – review & editing.

## Conflicts of interest

The authors declare that they have no known competing financial interests or personal relationships that could have influenced this study.

## Data Availability

No primary research results, software, or code have been included, and no new data were generated or analyzed as part of this review.
